# Effect of parathyroidectomy on serum inflammatory and metabolic dysfunction markers in patients with primary hyperparathyroidism

**DOI:** 10.20945/2359-4292-2024-0124

**Published:** 2024-11-06

**Authors:** Muzaffer Serdar Deniz, Nuriye Ozder, Omer Faik Ersoy, Zubeyde Ilke Narli

**Affiliations:** 1 Sincan Education and Research Hospital Department of Endocrinology Ankara Turkey Department of Endocrinology, Sincan Education and Research Hospital, Ankara, Turkey; 2 Karabük University Faculty of Medicine, Education and Research Hospital Department of General Surgery Karabük Turkey Department of General Surgery, Karabük University, Faculty of Medicine, Education and Research Hospital, Karabük, Turkey; 3 Karabük University Faculty of Medicine, Education and Research Hospital Department of Pathology Karabük Turkey Department of Pathology, Karabük University, Faculty of Medicine, Education and Research Hospital, Karabük, Turkey

**Keywords:** Primary hyperparathyroidism, parathyroidectomy, inflammation, TyG index, FIB-4 score

## Abstract

**Objective:**

This study analyzed systemic inflammatory changes reflected by hematologic and biochemical indices in patients with hyperparathyroidism (PHPT) after parathyroidectomy.

**Materials and methods:**

Retrospective study of 70 patients who underwent curative parathyroidectomy for PHPT treatment. Data on clinical presentation, biochemical assays, imaging studies, and postoperative outcomes were collected. Systemic inflammation was quantified using different indices, including the triglyceride-glucose (TyG) index, Fibrosis-4 (FIB-4) score, systemic immune-inflammation index (SII), monocyte-to-high-density lipoprotein cholesterol ratio (MHR), platelet-to-lymphocyte ratio (PLR), and platelet distribution width (PDW).

**Results:**

Significant pre-surgical to post-surgical decreases were observed in serum levels of mean normalized calcium (11 ± 0.65 mg/dL and 9.1 ± 0.42 mg/dL, respectively, p = 0.001) and parathyroid hormone (PTH) (235.5 ± 132.9 and 78.1 ± 60.5 ng/L, respectively, p = 0.001). The inflammatory indices changed substantially, with decreases in SII (from 564.8 ± 257.5 to 516.6 ± 201.1, p = 0.001) and PLR (from 143.0 ± 46.2 to 133.6 ± 38.6, p = 0.001). Additionally, PDW decreased from 52.8 ± 8.2% to 47.5 ± 9.3% (p = 0.001) and MHR increased from 7.19 ± 3.06 to 7.81 ± 3.13 (p = 0.001). No significant changes occurred in other inflammatory markers, including the TyG index (p = 0.431) and FIB-4 score (p = 0.401). Logistic regression analysis identified PDW (odds ratio [OR] 0.920, 95% confidence interval [CI] 0.879-0.963, p = 0.001) and PLR (OR 0.991, 95% CI 0.983-1, p = 0.042) as significant predictors of inflammation.

**Conclusions:**

Successful parathyroidectomy in patients with PHPT reduces systemic inflammation, as evidenced by decreased PDW and PLR. Our results indicate the importance of integrating PDW and PLR in the postoperative assessment of PHPT for monitoring inflammatory activity.

## INTRODUCTION

Primary hyperparathyroidism (PHPT) is an endocrine disorder marked by an excessive production of parathyroid hormone (PTH), disrupting calcium metabolism (1). Traditionally, the manifestations of PHPT are related to changes in hormone and calcium levels. However, this condition affects the body more broadly, particularly by promoting low-grade inflammation and increasing the risk of cardiovascular disease (2). Recent research highlights the significance of this systemic impact, pointing out that PHPT can intensify inflammatory processes throughout the body (3,4). Indeed, recent research evidence has contributed to the understanding of PHPT beyond its direct effects on calcium metabolism, including its lesser-characterized impact on inflammation (3,4). Integrating biomarker indices – such as the triglyceride-glucose (TyG) index, Fibrosis-4 (FIB-4) score, and systemic immune-inflammation index (SII), among others – into the diagnostic and evaluative framework offers a more nuanced understanding of the systemic changes in PHPT (5). These indices, calculated from routine blood tests, provide valuable insights into patients’ metabolic and inflammatory status, shedding light on the potential mechanisms through which PHPT exacerbates cardiovascular risk (6). Understanding and utilizing the links between PHPT and systemic inflammation through these scores may improve the comprehensive risk assessment of patients. This approach may also provide a deeper understanding of the relationship between inflammatory diseases and PTH.

The present study focused on PHPT and systemic inflammatory responses through hematologic inflammatory indices. These indices were meticulously selected for their established roles in reflecting various aspects of systemic inflammation and metabolic dysregulation, which are hypothesized to be altered in PHPT. These specific blood markers, which are frequently used to evaluate systemic inflammation, include the SII (7), the neutrophil-to-lymphocyte ratio (NLR) (8), the platelet-to-lymphocyte ratio (PLR) (9), red cell distribution width (RDW) (9), and platelet distribution width (PDW) (10). To further elucidate the systemic inflammatory landscape in PHPT, the study aimed to integrate a comprehensive panel of biomarker indices.

## MATERIALS AND METHODS

### Study design

This retrospective study was conducted at a tertiary care center from October 2021 to July 2023. We included 70 patients who underwent surgery for PHPT and demonstrated no disease persistence, as determined by postoperative analyses, establishing a postoperative period ≥ 6 months as appropriate for the evaluation. The patients’ follow-up period ranged from a minimum of 7 months to a maximum of 2 years.

The exclusion criteria included the presence of persistent PHPT, secondary or tertiary hyperparathyroidism, multiple endocrine neoplasia, any form of malignancy or solid organ tumors, inflammatory or hematologic diseases, use of drugs known to affect complete blood count indices, diabetes mellitus, active infections, renal or hepatic insufficiency, heart failure, pregnancy, and incomplete medical records.

### Clinical and demographic characteristics

Comprehensive demographic and clinical characteristics were collected from each participant, including age, sex, clinical manifestations of hyperparathyroidism, family history of parathyroid gland disease, and history of fragility fractures. Imaging findings, surgical procedure types, and histopathological examination results were recorded.

### Laboratory data related to primary hyperparathyroidism

Complications of PHPT were screened and recorded, including 24-hour urinary calcium levels, serum calcium levels, presence of kidney stones on ultrasound evaluation, and finding of osteoporosis on bone mineral densitometry. The outcome measures were based on inflammatory indices. Various tests were performed at least 6 months after surgery, including serum levels of normalized calcium, phosphate, magnesium, 25-hydroxyvitamin D (25-OHD), PTH, and alkaline phosphatase. The chloride-to-phosphate ratio and the glomerular filtration rate (GFR) were recorded.

### Parameters of systemic inflammatory response

Blood samples were collected after overnight fasting. Complete blood count (including differential), along with serum albumin, globulin, and lipid levels, were measured using standard laboratory techniques. We calculated each index using the provided formulas. The TyG index was calculated using the following formula: TyG index=Ln [fasting triglycerides(mg/dL)×fasting glucose(mg/dL)/2] , where Ln denotes the natural logarithm. The FIB-4 score was determined using the following equation: $\text{FIB-4 score}=\text{Age (years)×AST (U/L) /}[\text{ PLT}\left(10^{9}/\text{L}\right)\times \sqrt{\text{ ALT}}(\text{U}/\text{L})]$ , where PLT stands for platelet count, ALT stands for alanine aminotransferase, and AST stands for aspartate aminotransferase. The SII was computed by multiplying the platelet count by the neutrophil count (10^9^/L) and dividing the result by the lymphocyte count (10^9^/L). The systemic inflammation response index (SIRI) was derived by multiplying the neutrophil count (10^9^/L) with the monocyte count and dividing by the lymphocyte count (10^9^/L). The prognostic nutritional index (PNI) was calculated using the following formula: $\text{PNI}=10\times \text{serum albumin}(\text{g/dL})+0.005\times \text{ total lymphocyte count}\left({\text{per mm}}^{3}\right)$ . The monocyte-to-high-density lipoprotein cholesterol ratio (MHR) was calculated by dividing the monocyte count by the high-density lipoprotein (HDL) cholesterol level (mg/dL). The atherogenic index of plasma (AIP) was ascertained by taking the logarithm of the ratio between serum triglyceride and serum HDL (mg/dL). The albumin-to-globulin ratio (AGR) was established by dividing albumin by globulin (g/dL). The NLR was calculated by dividing the neutrophil count by the lymphocyte count (10^9^/L). The PLR was determined by dividing the platelet count by the lymphocyte count (10^9^/L).

### Statistical analysis

We used a comprehensive statistical approach to evaluate the outcomes following parathyroidectomy in patients with nonpersistent PHPT. The data were analyzed using SPSS version 25.0 (IBM Corp., Armonk, NY, USA). Continuous variables were expressed as mean ± standard deviation, and categorical variables were summarized as frequency (percentage). The normality of the distribution for continuous variables was assessed using the Shapiro-Wilk test. Comparisons between preoperative and postoperative parameters were conducted using Student's paired *t* test for normally distributed variables and the Wilcoxon signed-rank test for variables not following a normal distribution. To account for multiple comparisons, we applied the Bonferroni correction and the Benjamini-Hochberg method to adjust our significance thresholds. According to these strategies, the significance level (p value) was adjusted among all variables. The chi-square test or Fisher's exact test was applied to compare categorical data, as appropriate. Forward stepwise logistic regression was performed to examine the degrees of change in systemic inflammation markers. Variables entered into the model were selected based on their clinical relevance and importance in univariate analyses. Odds ratios (ORs) with 95% confidence intervals (CIs) were calculated to measure the strength of associations. A receiver operating characteristic (ROC) curve was used to evaluate the ability of PDW and PLR to discriminate changes in systemic inflammation markers. The area under the curve (AUC) was calculated to assess the overall accuracy of these biomarkers, and CIs provided a measure of precision. The optimal threshold values for PDW and PLR were determined by maximizing the Youden index and balancing sensitivity and specificity. Statistical correlations were analyzed using Spearman's correlation test. All tests were two-tailed, and p values < 0.05 were considered significant.

### Ethical considerations

The study was conducted in accordance with the ethical standards established by the Declaration of Helsinki. The study protocol was approved by our institution's Ethics Review Board (Date: November 3rd, 2023; Approval ID: 2023/1426). Before participation, all individuals were informed about the study's aims, procedures, and potential risks and benefits. Written informed consent was obtained from each participant, ensuring their voluntary involvement and their right to withdraw from the study at any point without consequences.

## RESULTS

### Demographic and clinical characteristics

The study participants had a mean age of 50.45 ± 12.3 years. In all, 59 (84%) were women and 11 (16%) were men. Overall, PHPT was symptomatic in 36% of the patients, with only a few of them reporting a positive family history of parathyroid disorders and 23% reporting kidney stones or nephrocalcinosis. The surgical management involved more frequently a minimally invasive parathyroidectomy (63%), highlighting a high prevalence of this approach (Table 1).

**Table 1:** Baseline demographic and clinical data of patients with primary hyperparathyroidism

Variables		Results*
Age, years		50.4 ± 12.3
Sex		
	Male	11 (16)
	Female	59 (84)
Primary hyperparathyroidism		
	Symptomatic	25 (36)
	Asymptomatic	45 (64)
Family history of parathyroid disease		
	No	67 (96)
	Yes	3 (4)
History of fragility fractures		
	No	70 (100)
	Yes	0
Kidney stone or nephrocalcinosis		
	No	54 (77)
	Yes	16 (23)
24-hour urinary calcium (mg/dL)		404.4 ± 186.55
Fractionated calcium		0.024
Bone mineral density		
	Osteoporosis	29 (41)
	Osteopenia	22 (31)
	Normal	19 (28)
Parathyroid adenoma weight (pathological specimen, mg)		778.9 ± 1348.48
Surgical procedure		
Minimally invasive parathyroidectomy		44 (63)
	Conventional bilateral neck exploration	4 (6)
	Parathyroid adenectomy + thyroid surgery	22 (31)

* The results are shown as mean ± standard deviation or frequency (percentage).

### Calcium metabolism indicators

Significant improvements were observed postoperatively across most parameters, indicating the effectiveness of the intervention. Specifically, the mean serum level of normalized calcium decreased from 11.0 ± 0.65 mg/dL preoperatively to 9.1 ± 0.42 mg/dL postoperatively (p = 0.001), returning to a normal range value. The mean serum phosphate level increased from 2.49 ± 0.51 mg/dL to 3.37 mg/dL ± 0.52 mg/dL (p = 0.001), whereas the chloride-to-phosphate ratio decreased from 45.34 ± 10.6 to 32.3 ± 5.27 (p = 0.001). The mean serum magnesium level had a slight reduction from 2.1 ± 0.2 mg/dL to 1.9 ± 0.2 mg/dL (p = 0.001), while the mean serum vitamin D level increased from 15.05 ± 7.2 ng/mL to 24.6 ± 12.4 ng/mL (p = 0.001). The mean PTH level decreased from 235.5 ± 132.9 ng/L preoperatively to 78.1 ± 60.5 ng/L postoperatively (p = 0.001), and the mean serum alkaline phosphatase decreased from 123.9 ± 95.4 U/L to 83.2 ± 27.1 U/L (p = 0.001). Notably, the GFR did not show a significant change postoperatively, with preoperative levels at 97.4 ± 19.4 mL/min/1.73 m² and postoperative levels at 95.4 ± 16.6 mL/min/1.73 m² (p = 0.361) (Table 2).

**Table 2:** Comparisons between preoperative and postoperative laboratory tests related to calcium metabolism in patients with primary hyperparathyroidism

Laboratory test	Preoperative	Postoperative	P value
Normalized calcium (serum; RR 8.7-10.4 mg/dL)	11.0 ± 0.657	9.1 ± 0.423	0.001
Phosphate (serum; RR 2.4-5.1 mg/dL)	2.49 ± 0.51	3.37 ± 0.52	0.001
Chloride-to-phosphate ratio	45.34 ± 10.6	32.3 ± 5.27	0.001
Magnesium (serum; RR 1.3-2.7 mg/dL)	2.1 ± 0.2	1.9 ± 0.2	0.001
Vitamin D (serum; RR 30-50 ng/mL)	15.05 + 7.2	24.6 + 12.4	0.001
Parathyroid hormone (serum; RR 15-65 ng/L)	235.5 ± 132.9	78.1 ± 60.5	0.001
Alkaline phosphatase (serum; RR 44-147 U/L)	123.9 ± 95.4	83.2 ± 27.1	0.001
Glomerular filtration rate (mL/min/1.73 m²)	97.4 ± 19.4	95.4 ± 16.6	0.361

The results are presented as mean ± standard deviation values. The values in parentheses in the first column represent reference ranges (RR) and units for each test result. The statistical analysis was done using Student's paired t test. All results showed significant differences with the intervention, except for the glomerular filtration rate, in which the p value indicated that the change was not significant.

### Inflammatory indices

We modified the significance levels using the Bonferroni correction and the Benjamini-Hochberg method to adjust for multiple comparisons. The significance level for the Bonferroni correction was changed to 0.0038 (*i.e.*, 0.05 divided by 13). Thus, in this analysis, only p values below 0.0038 were considered significant. P values for the Benjamini-Hochberg method were compared to their critical values and ranked. We could limit the percentage of false positives among the significant results by controlling the false discovery rate with this strategy. Within this analysis, p values of 0.0001 were considered significant.

The results indicated postoperative reductions in several markers, suggesting a decrease in systemic inflammation after surgery (Table 3). Specifically, the SII decreased from 564.8 ± 257.5 preoperatively to 516.6 ± 201.1 postoperatively (p = 0.0001), and the MHR increased from 7.19 ± 3.06 to 7.81 ± 3.13, respectively (p = 0.0001). Notable improvements were also observed in the PLR, which decreased from 143.0 ± 46.2 to 133.6 ± 38.6 (p = 0.0001), and in the PDW, which decreased from 52.8 ± 8.2% to 47.5 ± 9.3% (p = 0.0001). Other markers, including the TyG index, FIB-4 score, SIRI, PNI, AIP, and AGR showed no changes, indicating a lower influence from the intervention on these parameters. Of note, AGR showed a marginal but significant decrease (p = 0.043), while slight but significant changes were observed in the mean platelet volume (MPV; p = 0.037) and NLR (p = 0.035). The RDW showed a substantial increase, but it was not considered significant since its p value was excluded after adjustment for multiple comparisons.

**Table 3:** Comparison between preoperative and postoperative inflammatory markers in patients with primary hyperparathyroidism

Inflammatory markers	Preoperative	Postoperative	P value
TyG index (ratio)	8.77 ± 0.5	8.74 ± 0.55	0.431
FIB-4 score (ratio)	0.00083 ± 0.0003	0.00082 ± 0.0003	0.401
SII (ratio)	564.8 ± 257.5	516.6 ± 201.1	0.0001
SIRI (ratio)	738.1 ± 413.9	726.9 ± 281.9	0.292
PNI (ratio)	56.3 ± 4.4	56.1 ± 3.8	0.977
MHR (ratio)	7.19 ± 3.06	7.81 ± 3.13	0.0001
AIP (ratio)	0.44 ± 0.26	0.44 ± 0.25	0.524
AGR (ratio)	1.968 ± 0.58	1.957 ± 0.54	0.043
MPV (fL)	8.634 ± 0.96	8.637 ± 0.82	0.037
NLR (ratio)	2.066 ± 0.84	1.975 ± 0.66	0.035
PLR (ratio)	143.0 ± 46.2	133.6 ± 38.6	0.0001
PDW (%)	52.8 ± 8.2	47.5 ± 9.3	0.0001
RDW (%)	14.2 ± 1.4	14.4 ± 3.7	0.022

Abbreviations: AGR, albumin-to-globulin ratio; AIP, atherogenic index of plasma; FIB-4 score, Fibrosis-4 score; MHR, monocyte-to-high-density lipoprotein cholesterol ratio; MPV, mean platelet volume; NLR, neutrophil-to-lymphocyte ratio; PDW, platelet distribution width; PLR, platelet-to-lymphocyte ratio, PNI, prognostic nutritional index; RDW, red cell distribution width; SII, systemic immune-inflammation index; SIRI, systemic inflammation response index; TyG index, triglyceride-glucose index.

### Regression analysis

The final model from the forward stepwise regression analysis included two significant predictors of the outcome variable: PDW (OR 0.920, 95% CI 0.879-0.963, p = 0.0001) and PLR (OR 0.991, 95% CI 0.983-1, p = 0.042), with an R^2^ of 0.153. The analysis highlights the roles and implications of PDW and PLR in the model, providing insights into their contributions to the outcome variable.

### Receiver operating characteristic curve analysis

**Figure 1:**
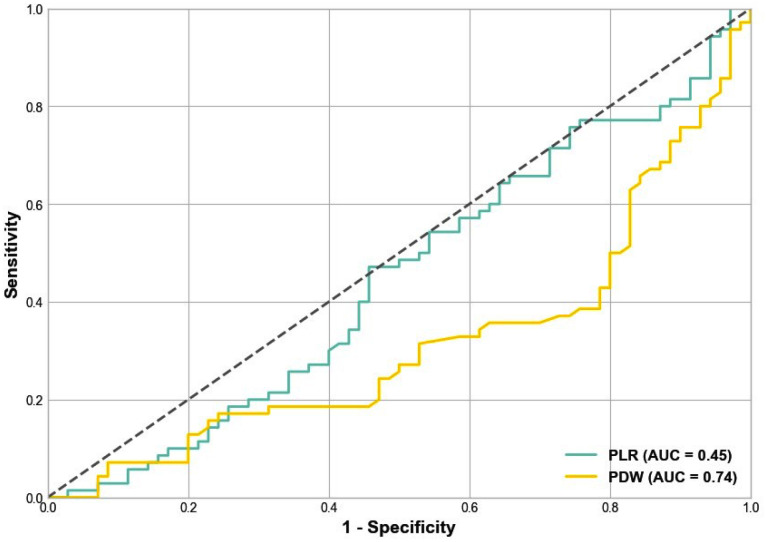
Receiver operating characteristic (ROC) curve showing the discriminatory ability of the platelet distribution width (PDW) and platelet-to-lymphocyte ratio (PLR) as systemic inflammatory markers in patients with primary hyperparathyroidism before and after curative parathyroidectomy. Abbreviation: AUC, area under the curve.

The PDW was the most discriminative variable, with an AUC of 0.745 (standard error 0.035, p = 0.0001) and CIs spanning from 0.626 to 0.844, indicating a discriminatory ability. The analysis, conducted under the nonparametric assumption, indicated that among the variables analyzed, PDW stood out with a significant discriminatory ability to differentiate between the groups studied. Using a cutoff value of 49.1 for PDW, the analysis demonstrated a sensitivity of 74.1% and a specificity of 72.3%. This indicates that PDW, at the specified threshold, effectively identifies the presence of the condition with a high true positive rate while also accurately excluding those without the condition at a comparable rate (Figure 1).

### Correlation analysis

Spearman's correlation analysis was performed to evaluate the relationships between the presence of kidney stones, serum calcium levels, osteoporosis, and preoperative inflammatory indices. The AGR demonstrated a strong negative and significant relationship with the presence of kidney stones (r = −0.375, p < 0.01). In contrast, a strong positive and significant relationship was observed between the presence of kidney stones and MHR (r = 0.241, p < 0.05). Additionally, a significant weak positive correlation was found between osteoporosis and MPV (r = 0.272, p = 0.027). No significant correlations were found with other indices.

## DISCUSSION

The findings of the present study present compelling evidence about the systemic inflammatory response and metabolic dysregulation in PHPT, elucidating the profound impact of the surgical intervention on these parameters. These findings advance the understanding of PHPT, highlighting it not only as a disorder of calcium metabolism but also as a condition with broad systemic implications, particularly inflammatory ones. They also emphasize the improvements in metabolic-related and calcium-related parameters, reinforcing the effectiveness of parathyroidectomy in restoring metabolic balance. The normalization of serum calcium, phosphate, vitamin D, and PTH levels underscores the surgery's critical role in correcting metabolic problems in PHPT. Parathyroidectomy – the surgical removal of the parathyroid glands – has been investigated as a therapeutic option to manage renal disease, aiming to control these metabolic disturbances (11,12). Researchers have suggested that early intervention through parathyroidectomy could potentially mitigate the progression of kidney disease and improve patients’ overall outcomes (13). Tassone and cols. conducted a study to evaluate the impact of parathyroidectomy on kidney function in patients with renal disease (14). Their research has provided valuable insights into the relationship between parathyroidectomy and renal health. Notably, the present study found significant changes in all calcium metabolism tests, but not in GFR. The reasons for this finding may be due to the presence of a GFR ≥ 60 mL/min/1.73 m² in most patients in the present study, the evaluation of kidney function tests in the early postoperative period, and the severity and duration of PHPT. Accumulating evidence suggests a potential link between elevated PTH and systemic inflammation. Patients diagnosed with PHPT show a notable upregulation in the expression of genes associated with inflammation (15). Rodent models subjected to a diet that induces hyperparathyroidism, marked by increased proinflammatory cytokines, also show similar upregulation (16). Novel hematologic indices reflecting systemic inflammation could help researchers understand the mechanisms of PHPT (17). Due to its cost-effectiveness and ease of measurement, routine complete blood count tests can be used to assess RDW, which reflects cell size heterogeneity (18). Cheng and cols. found that RDW correlates strongly with PTH and C-reactive protein (CRP) levels, further supporting the association between PTH and systemic inflammation (19).

Chronic inflammation is attributed to the proliferation of megakaryocytes, stimulated by the release of various immunologic mediators, including interleukin-6, in response to systemic inflammation (20). A study by Lam and cols. found a positive correlation between NLR, serum calcium, and PTH (21). Additionally, PLR has been identified as an affordable and efficient marker for the assessment of chronic inflammation, comparable to NLR (22). It has also been highlighted as superior to NLR in evaluating inflammation. Studies have evaluated PLR as an inflammatory marker for various chronic conditions (23). Recent findings indicated that parathyroidectomy reduces PLR in secondary hyperparathyroidism, suggesting the potential role of PLR in monitoring inflammatory responses after this surgery (24). Our study demonstrated a decrease in inflammatory markers after parathyroidectomy, including decreases in SII and NLR and improvements in PLR and PDW. This finding highlights the overall impact of PHPT on the body and the effectiveness of surgical treatment in reducing these inflammatory responses. The marked postoperative decrease in PDW is particularly notable and emphasizes its utility as a biomarker for the assessment of inflammation regression. It also highlights the role of surgery in alleviating the proinflammatory state associated with PHPT. The study's finding regarding the discriminatory ability of PDW, evidenced by the ROC curve, highlights a potential for monitoring changes in inflammatory markers after curative parathyroidectomy. The identification of PDW and PLR as determinants of changes in inflammatory markers through regression analysis further emphasizes their clinical relevance, providing a new perspective for evaluating PHPT and inflammation.

In various studies, MHR has been characterized as an emerging biomarker for inflammation in diverse systemic conditions (25,26). However, a study by Kızılgül and cols. comparing MHR in patients with PHPT *versus* controls found similar MHR results between the groups (27). We observed a significant postoperative increase in MHR in our study. The duration of preoperative exposure to PHPT could potentially explain this finding. Moreover, this increase may be temporary, and reevaluating this test over time to study its variability may provide more accurate insights. Jamialahmadi and cols. found that PTH levels did not change significantly or predict liver fibrosis and steatosis after bariatric surgery, despite changes in FIB-4 scores (28). Our study also showed no notable FIB-4 score change after parathyroidectomy. Rudman and cols. found no decrease in insulin resistance after curative parathyroidectomy in patients with PHPT (29). Similarly, our study showed no postoperative change in the insulin resistance equivalent TyG index. This persistence of specific markers postoperatively, such as the TyG index and FIB-4 score, indicates the complex and multifactorial nature of systemic inflammation in PHPT, suggesting that some inflammatory processes may remain unaffected by parathyroidectomy.

Persistent PHPT is defined as hypercalcemia occurring within 6 months from primary surgery for PHPT (30). Since the patients in the present study did not have persistent PHPT, evaluating them at ≥ 6 months postoperatively ensured that hypercalcemia was not a factor influencing the results. A period of 6 months is considered sufficient for the stabilization of the effects of surgery on inflammatory markers. This timeframe enables the acute inflammatory responses and any immediate postoperative alterations to dissipate, providing a more accurate assessment of the longer-term effects of surgery on inflammatory markers.

Spearman's correlation analysis found significant correlations between the presence of kidney stones and preoperative inflammatory indices. Specifically, there was a strong negative association with AGR and a strong positive relationship with MHR. In patients with PHPT, a lower AGR is associated with kidney stones. This implies that individuals with kidney stones typically exhibit a more prominent inflammatory response since a lower AGR usually indicates inflammation or malnourishment. Additionally, in our patients with PHPT, kidney stones were associated with higher MHR, an inflammatory marker. Therefore, kidney stones, being a common complication of PHPT, represent a more severe disease and systemic inflammation. Additionally, a significant weak positive correlation was found between MPV and osteoporosis (p = 0.027). This finding suggests that higher MPV may be associated with osteoporosis. This relationship can be explained by the fact that higher MPV may indicate increased platelet activation and inflammation, which could contribute to bone resorption and the development of osteoporosis.

Our study examining the relationship between systemic inflammatory markers and PHPT is the first in the literature to assess the inflammatory relationship between the TyG index and FIB-4 score with PHPT. Analyzing the systemic inflammatory condition in PHPT through hematologic indices was the most vital point of the study; however, the retrospective design of the study limits the ability to establish causality. Another limitation of our study is the potential influence of both the intake of vitamin D supplements and increased exposure to sunlight due to seasonal changes in vitamin D levels. However, we considered the increase in vitamin D level to be a component of the overall post-surgical improvement. Along with the other metabolic changes, the improved vitamin D levels may contribute to reducing the inflammatory response. Additionally, the study's single-center setting may affect the generalizability of the findings, indicating a need for multicenter studies to validate these results across diverse populations. The complexity of systemic inflammation in PHPT also indicates that other unmeasured factors may influence these markers, warranting further research to unravel the intricate web of interactions in PHPT pathophysiology.

In conclusion, in PHPT, a significant reduction in systemic inflammatory markers, particularly PDW and PLR, following curative parathyroidectomy suggests that this disease is not merely a disorder of calcium metabolism. These findings underscore the potential therapeutic benefits of parathyroidectomy in mitigating systemic inflammatory responses, thereby expanding our understanding of the broader health implications of PHPT. Our study should contribute to future research in elucidating the relationship between PHPT and inflammation.

## Data Availability

the data supporting the study findings are available from the corresponding author upon reasonable request.
